# Bayesian genomic models boost prediction accuracy for survival to *Streptococcus agalactiae* infection in Nile tilapia (*Oreochromus nilioticus*)

**DOI:** 10.1186/s12711-021-00629-y

**Published:** 2021-04-21

**Authors:** Rajesh Joshi, Anders Skaarud, Alejandro Tola Alvarez, Thomas Moen, Jørgen Ødegård

**Affiliations:** 1 GenoMar Genetics AS, Tjuvholmen allé 11, 0252 Oslo, Norway; 2 AquaGen AS, Sluppen, P.O. Box 1240, 7462 Trondheim, Norway

## Abstract

**Background:**

Streptococcosis is a major bacterial disease in Nile tilapia that is caused by *Streptococcus agalactiae* infection, and development of resistant strains of Nile tilapia represents a sustainable approach towards combating this disease. In this study, we performed a controlled disease trial on 120 full-sib families to (i) quantify and characterize the potential of genomic selection for survival to *S. agalactiae* infection in Nile tilapia, and (ii) identify the best genomic model and the optimal density of single nucleotide polymorphisms (SNPs) for this trait.

**Methods:**

In total, 40 fish per family (15 fish intraperitoneally injected and 25 fish as cohabitants) were used in the challenge test. Mortalities were recorded every 3 h for 35 days. After quality control, genotypes (50,690 SNPs) and phenotypes (0 for dead and 1 for alive) for 2472 cohabitant fish were available. Genetic parameters were obtained using various genomic selection models (genomic best linear unbiased prediction (GBLUP), BayesB, BayesC, BayesR and BayesS) and a traditional pedigree-based model (PBLUP). The pedigree-based analysis used a deep 17-generation pedigree. Prediction accuracy and bias were evaluated using five replicates of tenfold cross-validation. The genomic models were further analyzed using 10 subsets of SNPs at different densities to explore the effect of pruning and SNP density on predictive accuracy.

**Results:**

Moderate estimates of heritabilities ranging from 0.15 ± 0.03 to 0.26 ± 0.05 were obtained with the different models. Compared to a pedigree-based model, GBLUP (using all the SNPs) increased prediction accuracy by 15.4%. Furthermore, use of the most appropriate Bayesian genomic selection model and SNP density increased the prediction accuracy up to 71%. The 40 to 50 SNPs with non-zero effects were consistent for all BayesB, BayesC and BayesS models with respect to marker id and/or marker locations.

**Conclusions:**

These results demonstrate the potential of genomic selection for survival to *S. agalactiae* infection in Nile tilapia. Compared to the PBLUP and GBLUP models, Bayesian genomic models were found to boost the prediction accuracy significantly.

**Supplementary Information:**

The online version contains supplementary material available at 10.1186/s12711-021-00629-y.

## Background

Nile tilapia is an important aquaculture species because of its wide range of trophic and ecological adaptations, which allows it to be farmed in different environments around the world. Farming of Nile tilapia is one of the fastest-growing aquaculture activities in more than 120 countries and, in 2017, it accounted for 5.3% of the global aquaculture production. Nile tilapia ranks 4th among the top ten aquaculture species in terms of both production quantity and value [1, 2]. For the last three decades, the tilapia sector has seen a rapid increase (11% per year) in global production, which is higher than the average growth for other aquaculture species [3, 4]. Intensification of tilapia farming results in high stocking densities and poorer water quality which, coupled with sub-optimal temperatures and mishandling of the fish and water, are a cause of stress on the animals throughout the growing period [5]. Because of these conditions, farmed tilapia are more exposed to various bacterial, viral, fungal, and parasitic diseases than wild tilapia [6].

Streptococcosis is a disease caused by the pathogens *Streptococcus agalactiae* and *Streptococcus iniae* and is considered one of the most significant bacterial diseases in Nile tilapia based on socio-economic impact and zoonotic potential [7]. Of these two Streptococcus species, *S*. *agalactiae* is the most prevalent [8] and causes significant morbidity and mortality [9], with mortality rates over 50% for acute infections [10]. In 2019, the losses were estimated to reach up to 1.5 billion USD per year in China alone, the largest producer of tilapia [11]. Symptoms of Streptococcosis are lethargy, erratic swimming, hyper-pigmentation of the skin, exophthalmia with haemorrhagic eyes, splenomegaly, abdominal distension, and diffused haemorrhage in the operculum, around the mouth and anus, and at the base of the fins [12–14].

Various short-term strategies to contain *S. agalactiae* using antibiotics and vaccines are deployed around the world [15–17], each with their own deficiencies. For example, use of antibiotics is expensive and complex because of the long withdrawal period and the increasing concerns about anti-microbial resistance in both fish and humans [6, 15, 18]. Development of resistant tilapia strains represents one of the long-term sustainable strategies to control this disease [19]. Selection against infectious diseases has been widely and successfully implemented in aquaculture species such as Atlantic salmon [20–22], and has motivated similar developments in various species, including against *S. agalactiae* in Nile tilapia [23–26].

Genetic selection for survival to *S. agalactiae* infection in GST® Nile tilapia using classical selection methods, in which resistance is assayed in siblings of the selection candidates, has resulted in strains/products that have a nearly two-thirds lower risk of mortality compared to the non-selected line [26]. Classical selection through sib-testing allows only the between-family variation to be used, which limits the accuracy of selection [27]. In addition, with restrictions on inbreeding, selection based on sib-testing hampers the rate of genetic gain because of limits on selection of closely-related individuals. The use of genomic selection methods has the potential to increase the rate of genetic improvement by allowing the use of within-family genetic variation, thereby increasing the accuracy of selection [27, 28]. Previous studies [29–33] have shown the benefits of using genomic selection for commercially important traits in Nile tilapia.

Our objectives were to: (i) to evaluate and characterize the potential of genomic selection for *S. agalactiae* control in Nile tilapia; (ii) explore the effect of pruning and density of single nucleotide polymorphisms (SNPs) on the prediction accuracy of different models for survival to *S. agalactiae* infection in Nile tilapia; and (iii) identify the best genomic prediction model for implementation of genomic selection for survival to *S. agalactiae* infection in Nile tilapia.

## Methods

### Study population

The breeding program for GenoMar Supreme Tilapia (GST®) in the Philippines is a continuation of the Genetically Improved Farmed Tilapia (GIFT) program at the commercial level. The genetic base of GIFT was formed by the systematic admixture of eight wild and commercial strains of Nile tilapia [34]. GenoMar bought generation 10 of the GIFT strain and since then has bred this line for growth, fillet yield, and robustness [29].

Each generation of the GST® line used in this study consists of 250 families distributed across eight batches that follow a revolving breeding scheme [30]. The families within each batch are created by mating the selected parents in a 1:1 mating design, where one male and one female are placed in a small breeding hapa. After mating, eggs are collected and the families are kept separate until the challenge test. The fish used in this study originated from four batches of generation 27 of the GST® strain.

### Challenge test

A controlled disease challenge test was performed using the *Streptococcus agalactiae Ib* strain. Overall, 108 full-sib families from generation 27 of the GST® strain were challenged in four batches. The dose (LD50) used was based on a previous study [35], in which it caused a 50% mortality rate in intra-peritoneal (IP)-challenged fish (i.e. injection of the pathogen directly in the intra-peritoneal region of the fish). Before the challenge test, each family was kept in separate tanks until individuals reached an average weight of 8 to10 g. A random 40 fish per family were tagged for the challenge test, of which a random 15 fish were IP injected (0.05 mL of bacterial strain) and then placed into a family tank along with the remaining 25 fish, which were used as cohabitants. Mortalities were monitored every 3 h, with the identity of each dead fish recorded and a fin clip collected. After 35 days, no mortalities had occurred for three consecutive days and the experiment was terminated by euthanizing the surviving fish and collecting their identification and fin clips. The survival phenotype at the end of the experiment was coded as a binary trait: 0 for the fish that died during the experiment and 1 for those that survived to 35 days.

### Genotypes

To reduce genotyping costs, only the 2700 cohabitant fish were genotyped since they were considered to best mimic the conditions of a disease outbreak in farm conditions. Genomic DNA was isolated from fin clip samples and genotyping was performed using the Onil50 Affymetrix Axiom Custom Array [36]. The genotypes were subjected to several quality control (QC) filters. Only SNPs identified as PolyHighResolution and NoMinorHomozygous by Affymetrix’s Axiom Analysis Suite software [37], were selected. In addition, individuals with a genotype call rate lower than 90% were removed. After quality control, 2472 animals genotyped for 50,690 SNPs were available for analysis (see Additional file 1: Table S1).

### Statistical analysis

#### Pedigree best linear unbiased prediction (PBLUP)

DMU [38] was used to fit the following univariate mixed linear PBLUP model using restricted maximum likelihood (REML) analysis to estimate the variance components and breeding values:

$$\mathbf{y}=\mathbf{X}{\varvec{\upbeta}}+\mathbf{Z}\mathbf{a}+\mathbf{e},$$

where $\mathbf{y}$ is the vector of phenotypes coded as 0 for dead fish and 1 for surviving fish after the challenge test, ${\varvec{\upbeta}}$ is a vector of fixed batch effects (4 levels), $\mathbf{a}$ is a vector of random additive genetic effects, $\mathbf{e}$ is a vector of random residuals, and $\mathbf{X}$ and $\mathbf{Z}$ are design matrices. Vectors $\mathbf{a}$ and $\mathbf{e}$ were assumed to be multivariate normally distributed with a mean of zero and variances:

$$\text{Var}\left[{\begin{array}{c}\mathbf{a}\\ \mathbf{e}\end{array}}_{ }\right]=\left[\begin{array}{cc}\mathbf{A}{\sigma }_{A}^{2}& 0\\ 0& \mathbf{I}{\sigma }_{E}^{2}\end{array}\right],$$

where ${\sigma }_{A}^{2}$ and ${\sigma }_{E}^{2}$ are the additive genetic variance and error variance, respectively, $\mathbf{A}$ is the numerator relationship matrix, and $\mathbf{I}$ is an identity matrix of appropriate size. The numerator relationship matrix was calculated using a 17-generation pedigree in the breeding nucleus, which was established based on parentage assignment using microsatellites, as described in [39]. The phenotypic variance was calculated as ${\sigma }_{P}^{2}={\sigma }_{A}^{2}+{\sigma }_{E}^{2}$, and the narrow sense heritability (${h}^{2}$) was calculated ${\sigma }_{A}^{2}/{\sigma }_{P}^{2}$.

#### Genomic models

Genomic BLUP (GBLUP) is the most commonly used genomic model for routine genetic evaluation because of its simplicity and low computation cost. The approach has been shown to be statistically equivalent to marker-effects BLUP model, SNP-BLUP [40–43]. The distinction between GBLUP and SNP-BLUP is that GBLUP estimates genomic estimated breeding values (GEBV) directly, while SNP-BLUP estimates marker effects. The underlying assumption for both GBLUP and SNP-BLUP is a normal prior with the same variance for all marker effects, with the relative contribution of each marker to the prior depending on its minor allele frequency [42, 44, 45].

The model fitted for GBLUP is the same as that for PBLUP, except that the pedigree relationship matrix $\mathbf{A}$ is replaced by the genomic relationship matrix $\mathbf{G}$, which was constructed as follows [45]:

$$\mathbf{G}=\frac{\mathbf{M}{\mathbf{M}}^{\mathbf{^{\prime}}}}{\sum 2{p}_{i}(1-{p}_{i})},$$

where $\mathbf{M}$ is a centered marker matrix, the sum in the denominator is over all loci and ${p}_{i}$ is the allelic frequency at locus $i$.

#### Bayesian models

The assumption that all the markers explain the same amount of the variance in GBLUP may not be suitable for traits that are less polygenic or controlled by some loci with major effects  [48, 49]. Hence, GCTB2.0  [50] was used to fit four genomic Bayesian mixed models: BayesB [51], BayesC [52], BayesR [46], and BayesS [50] . The following marker-effects model was fitted:

$$\mathbf{y}=\mathbf{X}{\varvec{\upbeta}}+\mathbf{M}\mathbf{Z}\mathbf{s}+\mathbf{e},$$

where $\mathbf{Z}$ is a diagonal matrix with diagonal elements 0/1 for SNPs excluded/included in the model, $\mathbf{s}$ is a vector of allele substitution effects for each SNP, and $\mathbf{M}$ is the (centered) marker matrix. All other parameters were as described for the PBLUP model. All Bayesian models used are variable selection models, with a prior that assumes that many SNPs have zero effect and the genetic variation is explained by a subset of markers (see  [47] for detailed explanation). The prior distribution of the variances of $\mathbf{s}$ differs among the Bayesian models, as follows.

*BayesB*: Each SNP effect is assumed to have an independent and identically-distributed mixture prior of a scaled $\text{t}$-distribution $\text{t}(0,{\uptau }^{2},\upupsilon )$ with probability $\uppi$ and a point mass at zero with probability $1-\uppi$, where ${\uptau }^{2}$ and $\upupsilon$ are prior hyperparameters  [51, 52].

*BayesC*: Each SNP effect is assumed to have an independent and identically-distributed mixture prior of a normal distribution that has mean 0 and variance ${\upsigma }^{2}$ with probability $\uppi$ and a point mass at zero with probability $1-\uppi$ [52].

*BayesR*: Each SNP effect is assumed to have an independent and identically-distributed mixture prior of multiple normal distributions that have mean 0 and variance ${\upgamma }_{\text{k}}{\upsigma }_{\text{k}}^{2}$ with probability ${\uppi }_{\text{k}}$ and a point mass at zero with probability $1-{\Sigma }_{\text{k}}{\uppi }_{\text{k}}$, where ${\upgamma }_{\text{k}}$ is a given constant  [46].

*BayesS*: BayesS is similar to BayesC but the variance of SNP effects (for SNPs with non-zero effects) is related to minor allele frequency ($\text{p}$) through a parameter $\text{S}$, i.e. ${\upsigma }_{\text{j}}^{2}={[2{\text{p}}_{\text{j}}\left(1-{\text{p}}_{\text{j}}\right)]}^{\text{S}}{\upsigma }^{2}$  [50].

Model parameters and SNP effects in the Bayesian models were estimated using the Markov chain Monte Carlo (MCMC) sampling algorithm implemented in the GCTB2.01 software [48]. The default parameters were used to determine the length of the MCMC (21,000 cycles), the number of cycles for burn-in (the initial 1000 cycles were discarded), and the thinning interval (10). The value of $\uppi$ was estimated from the data using the default starting value of 0.05 (–pi 0.05). The default starting value of 0.5 was used for the sampling of SNP-based heritability (–hsq 0.5). Convergence of the MCMC was verified by Geweke-Brooks plots [53] using R [54]. Because marker-based models estimate SNP effects, PLINKv1.90b6.7 [55] was used to calculate GEBV by summing the product of the effect estimate and genotype (0/1/2) for each SNP for each individual.

### Cross-validation and prediction accuracy

The prediction accuracy of the models was estimated based on five replicates of a tenfold cross-validation scheme. In tenfold cross-validation, the phenotypes of 10% of the animals are masked and then estimated using the phenotypes and genotypes of the remaining 90% animals. The dataset of genotyped animals with phenotypes was randomly divided into 10 subsets, predicting one subset (n = 247 or 248) at a time and using the phenotypes of the remaining nine subsets (n = 2224 or 2225) for training.

The predictive ability of the models was calculated as the Pearson’s correlation between predicted GEBV (or EBV in the case of PBLUP) in one replicate using the complete dataset and phenotypes adjusted for the fixed effects using the complete dataset. Results were averaged over the five replicates. The mean correlation value was converted into the expected prediction accuracy by dividing by the square root of the estimate of heritability based on PBLUP (0.15). The standard error of prediction accuracy was calculated [56] as:

$$\frac{1-{prediction\,accuracy}^{2}}{\sqrt{No. \,of\, validation\, animals-1}}.$$

The regression coefficient of phenotypes adjusted for fixed effects on (G)EBV was used to assess the bias of the predictions. The mean and standard error of the regression coefficient were calculated from the five replicates. A regression coefficient of 1 indicates unbiased prediction, whereas values lower or higher than 1 indicate inflation and deflation of (G)EBV, respectively.

### Low-density SNP subsets

Ten subsets of the SNP panel were created as described in the following, to assess the potential of using a lower density SNP set. For each SNP subset, prediction accuracies and biases were determined using the statistical analyses with the genomic models and cross-validations described in the previous sections.

Generally, selection of SNPs for a low-density chip should aim at including at least one SNP that is in strong linkage disequilibrium (LD) with each QTL for the trait. For this purpose, an LD-based SNP pruning method was used to select different subsets of SNPs. The LD between each pair of SNPs was calculated as the squared coefficient of correlation (r^2^) between 0/1/2 genotypes.

The set with all 50,690 SNPs will be referred to as the “All SNPs” panel. In the “only LG” subset, only SNPs assigned to linkage groups [36] were used, i.e. SNPs that are not assigned to a linkage group and those assigned to the mitochondrial genome [57] were removed. The SNPs in the “only LG” subset were pruned based on different LD value thresholds, using PLINKv1.90b6.7 [58]. The thresholds used for pruning were r^2^ $\le$ 0.1, 0.2, 0.3, 0.4, 0.5, 0.6, 0.7, 0.8 and 0.9 and the subsets are named based on these thresholds. For example: in subset “LD0.1”, only one SNP in a pair or group of SNPs that had r^2^ values higher than 0.1 was kept. The number of SNPs available for analysis for each subset is in Table 1.

**Table 1:** Estimates of heritability for different models and SNP densities for *Streptococcus* resistance in Nile tilapia

Sub-set	Number of SNPs	GBLUP		BayesB		BayesC		BayesR		BayesS	
		${h}^{2}$	se	${h}^{2}$	se	${h}^{2}$	se	${h}^{2}$	se	${h}^{2}$	se
LD0.1	589	0.09	0.02	0.06	0.01	0.09	0.02	0.10	0.02	0.10	0.02
LD0.2	1544	0.16	0.03	0.17	0.02	0.18	0.02	0.17	0.02	0.18	0.02
LD0.3	3384	0.16	0.03	0.17	0.02	0.18	0.02	0.17	0.02	0.19	0.02
LD0.4	6229	0.19	0.03	0.22	0.02	0.21	0.03	0.20	0.03	0.23	0.02
LD0.5	10,004	0.19	0.03	0.22	0.03	0.21	0.03	0.19	0.03	0.23	0.02
LD0.6	14,563	0.19	0.03	0.22	0.03	0.21	0.03	0.19	0.03	0.23	0.03
LD0.7	19,873	0.19	0.03	0.25	0.03	0.23	0.03	0.19	0.03	0.23	0.03
LD0.8	25,693	0.18	0.03	0.24	0.03	0.25	0.02	0.19	0.03	0.23	0.03
LD0.9	32,077	0.17	0.03	0.23	0.03	0.26	0.03	0.17	0.02	0.23	0.03
Only LG	48,871	0.15	0.03	0.26	0.03	0.27	0.03	0.15	0.02	0.26	0.02
All SNPs	50,690	0.15	0.03	0.26	0.03	0.25	0.05	0.26	0.05	0.24	0.04

The rows LD0.1 to LD0.9 represent the subsets obtained after pruning the SNPs based on LD values. For example: in subset “LD0.1” only one SNP in a pair or group of SNPs that had an LD value higher than 0.1 was kept

se = standard error of the heritability ($${h}^{2})$$

## Results and discussion

To our knowledge, this is the first study that uses genomic data to investigate genetic resistance to any disease in Nile tilapia and to quantify and characterize the potential of genomic selection to control *S. agalactiae* in Nile tilapia.

**Fig. 1:**
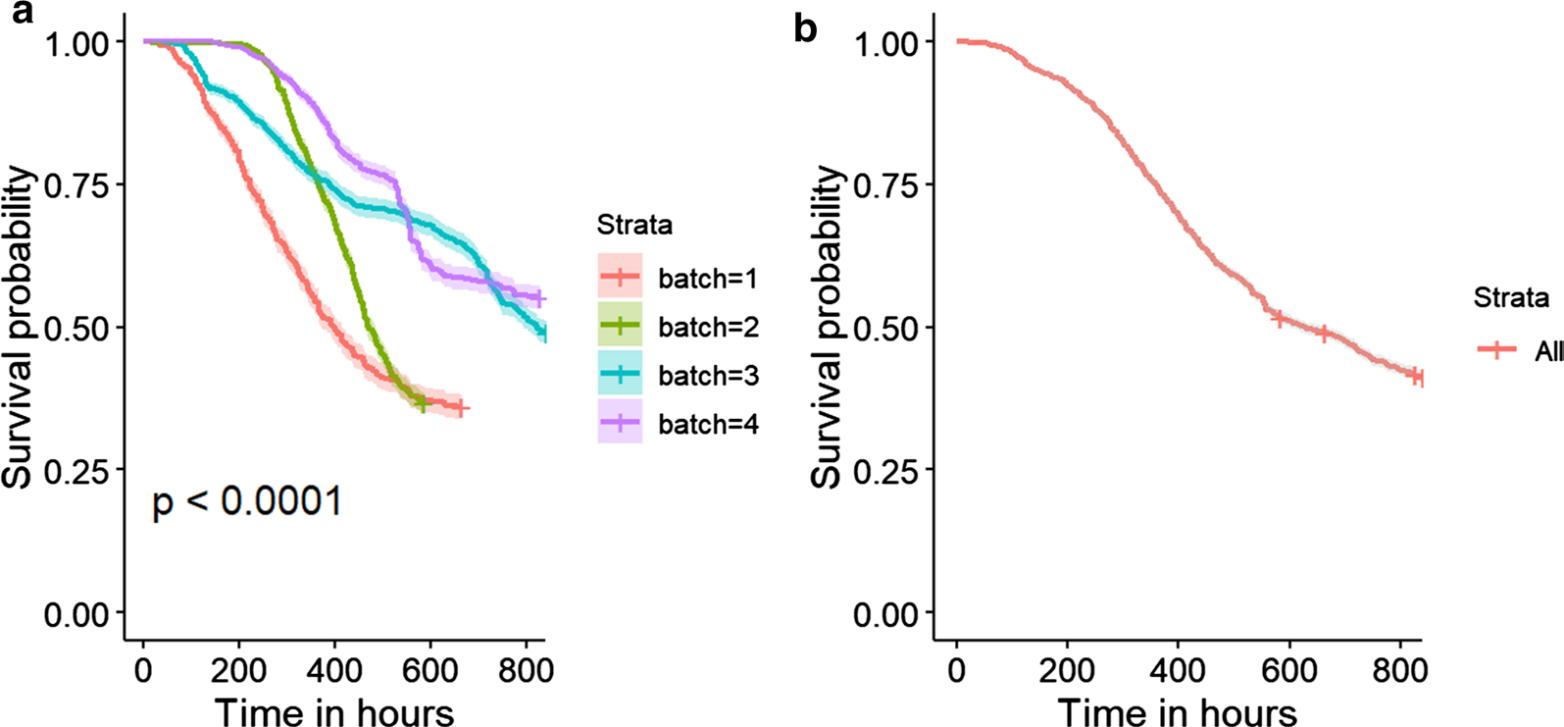
Kaplan–Meier curves for survival of the fish in the challenge test **a** by batch and **b** in the entire population

Average mortality during the challenge test was 60.2% and ranged from 49.5 to 67% across the batches. The Kaplan–Meier curves [59] in Fig. 1 show the cumulative mortality over the test period (Fig. 1). Although mortality was recorded as a binary phenotype, a linear model was used in the analyses. While a threshold model would have been theoretically more appropriate, several studies have shown good agreement between breeding values that are estimated using these two models [60–62].

### Genetic parameters

Heritability estimates for survival to *Streptococcus* infection in Nile tilapia using different models and SNP densities are in Table 1. A summary of the posterior mean of the key model parameters is in Additional file 2: Table S2. Using PBLUP, the estimated heritability was 0.15 ± 0.02, which is similar to that reported by Sukhavachana et al. [24] and slightly lower than the estimates reported by Shoemaker et al. [23]. The genomic models resulted in similar estimates of heritability for the “All SNPs” dataset, ranging from 0.15 ± 0.03 to 0.26 ± 0.05 (Table 1). The moderate estimates of heritability indicate that the Nile tilapia breeding industry can benefit from the application of selective breeding for survival to *Streptococcus* infection.

Differences in heritabilities estimated using different SNP densities are due to different amounts of genetic variation being captured by the SNPs. Heritability estimates using the LD0.1 SNP-set were significantly different from the estimates obtained by using the other SNP subsets (Table 1). As expected, very low SNP densities (i.e. using LD0.1 SNP-set) resulted in lower heritabilities for all genomic models (Table 1), because fewer SNPs are less likely to capture the majority of the genetic variance across the genome. Surprisingly, increasing the marker density had a different effect on estimates of heritability for GBLUP versus Bayesian models, potentially due to the presence of major QTL. For GBLUP, increasing the marker density should theoretically enable the model to more efficiently capture the majority of the genetic variance, resulting in higher heritability estimates. However, in our data, the heritability was highest (0.19) when the moderately pruned SNP-sets (LD0.4 to LD0.7) were used, compared to the highest SNP densities (${h}^{2}$ = 0.15 for only LG / all SNPs subsets), although these estimates were not significantly different based on standard errors.

For the Bayesian models, reducing the number of SNPs by pruning, generally reduced the estimate of heritability compared to using all SNPs. For the Bayesian models, the prior genetic variance for a DNA segment is no longer a function of the total number of SNPs (i.e. for GBLUP) but rather depends on the number of SNPs that have an effect (and for some of the models, the variance of their effect). Hence, it is likely that these models will be able to capture loci with a large effect (provided that the data include SNPs in LD with the QTL). Furthermore, compared to GBLUP the Bayesian models allow greater variance for some SNPs, which results in less shrinkage of the estimates [63].

Excluding the mitochondrial SNPs and the SNPs not assigned to any LG (“All SNPs” vs “Only LG”) either increased or did not affect the estimate of heritability but the change was not significant for most models, except for BayesR. For the BayesR model, a large decrease in the heritability estimate was observed using the “Only LG” subset of SNPs, compared to “All SNPs”.

### Prediction accuracy

**Fig. 2:**
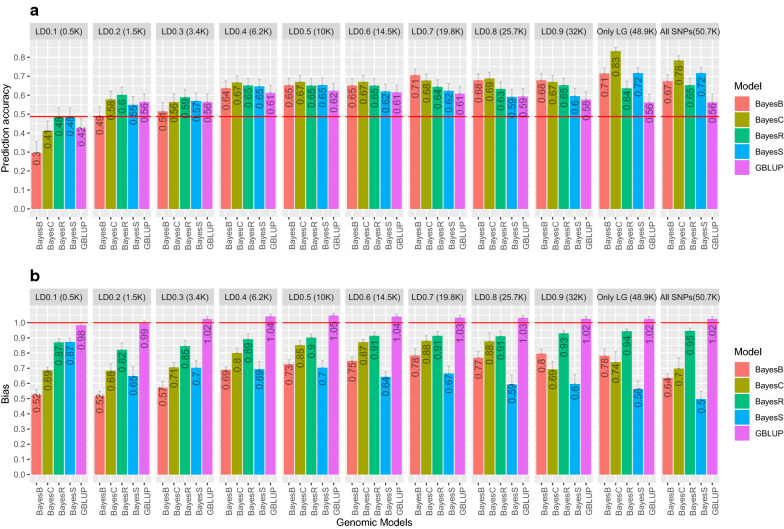
Prediction accuracy and bias of estimated breeding values for *S. agalactiae* resistance using different models. **a** Prediction accuracy. The red horizontal line represents the prediction accuracy using the PBLUP model (0.49). **b** Prediction bias. The red horizontal line represents the prediction bias using the PBLUP model (1). The grey lines in the bar charts represent standard errors

Prediction accuracies based on tenfold random cross-validation for different models and SNP subsets are shown in Fig. 2a. Prediction accuracy was estimated to be 0.49 using the PBLUP model. Genomic models were found to increase prediction accuracy compared to the PBLUP model for almost all SNP subsets (Fig. 2a), except the LD0.1 SNP subset. The relative increases in prediction accuracy for genomic models compared to PBLUP are in Additional file 3: Figure S1. It should be noted that in the cross-validation approach used here, the prediction accuracy of breeding values is estimated assuming a certain heritability, which here was calculated based on pedigree data. Hence, if the assumed heritability is set to a too low value, the estimated accuracies will be overestimated (i.e., the Bayesian models estimate a higher heritability), but the relative performance of the different models will not be affected.

### Accuracy with GBLUP

For the “All SNPs” subset, prediction accuracy of the GBLUP model (0.56) was 15.4% higher than with the PBLUP model (0.49). This increase in prediction accuracy by replacing the pedigree-based numerator relationship matrix by the genomic relationship matrix has been well documented in various species (e.g.  [49, 64, 66]). This is because the GBLUP model can use both within- and between-family genetic variation for traits that cannot be measured directly on the selection candidates, such as disease resistance [27, 28]. The PBLUP model, in contrast, can use only between-family genetic variation for such traits.

#### Accuracy with Bayesian models

**Fig. 3:**
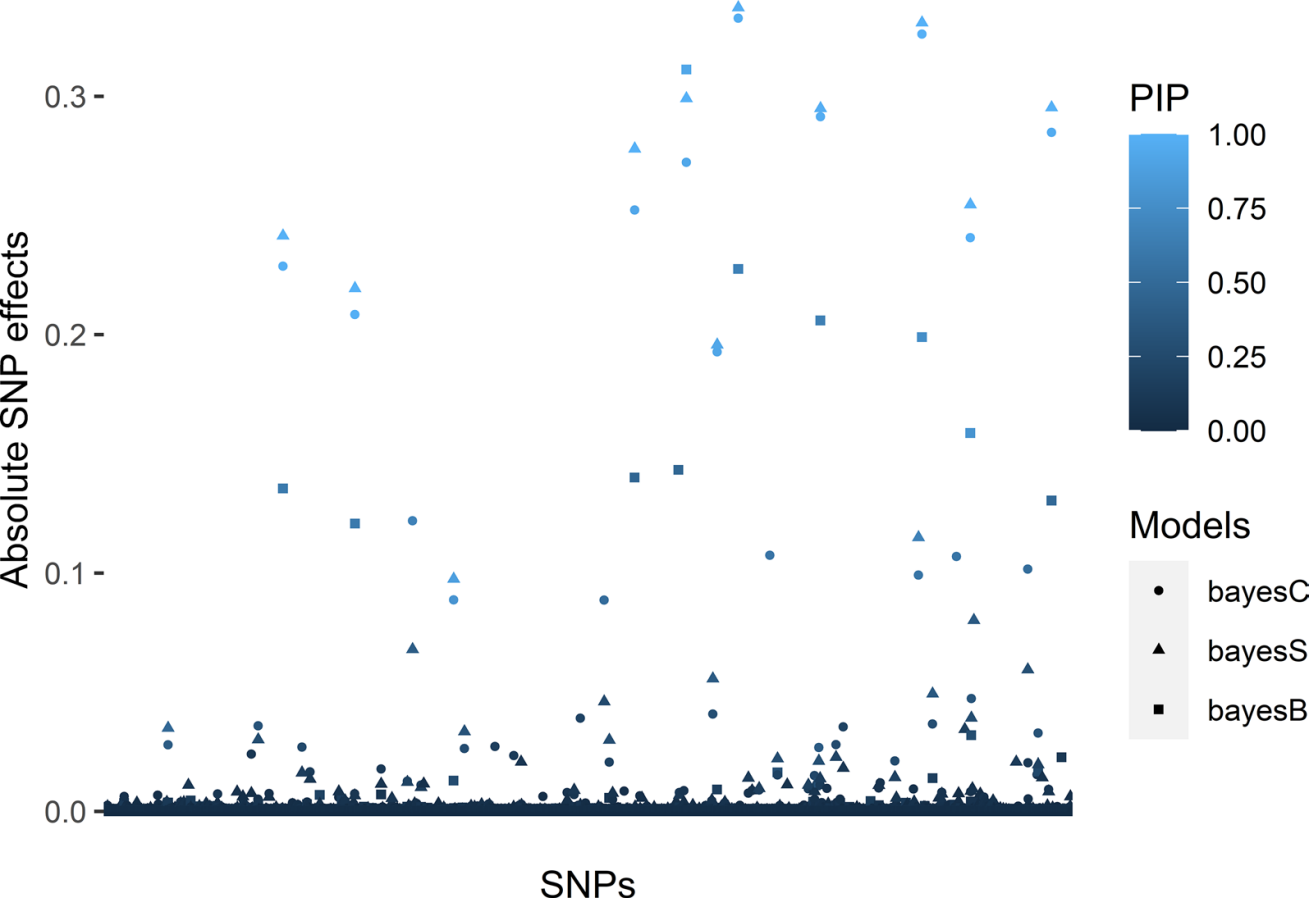
Absolute values of the estimates of SNP effects and posterior inclusion probabilities of the SNPs (PIP) obtained using different Bayesian models using the “Only LG” subset. The shape of the points denotes different Bayesian models and the intensity of the colour of the points denotes the posterior inclusion probabilities of the SNPs (the darker the colour, the lower the value)

For the “All SNPs” subset, prediction accuracy of the Bayesian models was higher than that of the GBLUP model (Fig. 2a), with the BayesC model resulting in the highest prediction accuracy (0.78), followed by BayesS (0.72), BayesB (0.67), and BayesR (0.65). The accuracy of genomic prediction depends on the model applied, which is representative of the architecture of the trait. Depending on the genetic architecture of the traits, one or the other class of models may perform better, because of their prior assumption about the SNP effects. Bayesian models assume that the genetic variation is explained by a small fraction of the SNPs, which may have an advantage over the GBLUP model when the architecture of the trait is (partly or entirely) controlled by a number of major QTL  [67], for example for some disease resistance traits that are controlled by a few major QTL (e.g.  [67-70]). However, if the architecture of the trait is polygenic, GBLUP models may be equally accurate, or in some cases even superior to the Bayesian models  [67]. In our case, the higher accuracy of the Bayesian models may indicate that the trait is controlled by a limited number of major QTL, which is further supported by the results in Fig. 3. Similar to *S. agalactiae*, it has been reported that resistance to another strain of *Streptococcus spp.*, *S. iniae*, is also affected by a major QTL  [71]. Thus, Bayesian models can result in higher accuracies of genomic prediction for survival to Streptococcosis.

### Effect of SNP density on prediction accuracy

For almost all the models used here, we found that the prediction accuracy increased or remained constant when only the SNPs mapped to linkage groups were used and when mitochondrial or unmapped SNPs were removed (Fig. 2). However, simulation studies have shown that prediction accuracy decreases gradually as the SNP density decreases  [72, 73], a result that has also been observed with real data [65, 75]. Mitochondrial DNA is haploid and exclusively maternally inherited. However, the standard SNP calling pipeline is adapted to diploid SNPs, which may affect the genotype quality of mitochondrial SNPs and thus may explain why their removal increases the prediction accuracy.

By pruning SNP density based on LD, the prediction accuracy of GBLUP gradually increased and peaked at ~ 10 K SNPs (LD0.5) (Fig. 2). For BayesB, BayesC and BayesS, pruning did not have a positive effect and prediction accuracies were highest with the highest density of 48.9 K SNPs (“Only LG” SNP subset). However, for BayesR, pruning had little effect up to 6.2 K SNPs (LD0.4), and the prediction accuracy was reduced at lower densities (≤ 3.4 K SNPs). Across models and densities, the BayesC model using the 48.9K SNP panel resulted in the highest prediction accuracy. Genomic models with as few as 600 SNPs (LD0.1) were found to achieve comparable prediction accuracies as PBLUP (Fig. 2).

For the Bayesian models, a smaller subset of the SNPs is actually used to model the genetic variance for each cycle of the MCMC. As expected, the value of $\uppi$ increased with decreasing SNP density in almost all cases for the Bayes B, C and S models (see Additional file 2: Table S2). In contrast, BayesR uses four $\uppi$ values that sum to 1 and the number of SNPs having a non-zero effect was predicted to be larger with BayesR than with the other Bayesian models. Furthermore, the SNPs with a non-zero effect overlapped between the Bayesian models (Fig. 3). In the “Only LG” SNP subset, BayesB, BayesC and BayesS models consistently (with respect to marker id and/or marker locations) included 40 to 50 SNPs with non-zero effects. Thus, the trait, survival to *S. agalactiae* infection, is controlled by several large QTL and our results obtained with the required minimum number of SNPs likely do not generalise to more polygenic traits.

### Prediction bias

Prediction biases obtained by tenfold random cross-validation and the different models and SNP subsets are shown in Fig. 2b. The bias was lowest with the GBLUP models for all datasets, while the Bayesian genomic models resulted in inflated GEBV, as evidenced by the regression coefficients of predicted phenotypes on GEBV that were lower than 1. Among the Bayesian models, GEBV were most inflated for BayesS and least inflated for BayesR for almost all subsets of SNP densities. For GBLUP, the GEBV were slightly inflated when the number of SNPs was smaller than the number of animals (n = 2472) and the inflation increased slightly as marker density decreased (i.e. the inflation was greater for the LD0.1 than for the LD0.2 SNP subsets).

In Nile tilapia, selection takes place in a single generation of individuals and, as a result bias does not affect the ranking of selection candidates and is not a major concern  [74]. However, for other purposes such as estimation of genetic gain, bias can influence the results.

## Conclusions

Our results demonstrate the potential of genomic selection for survival to *S. agalactiae* infection in Nile tilapia breeding programs. Using a BayesC model and a 48.9K SNP subset, the prediction accuracy was 71% higher than using a pedigree-based model, but resulted in more biased estimated breeding values. However, genomic models with as few as 600 SNPs can achieve comparable prediction accuracies as PBLUP. Provided all management practices remain constant, the potential increase in genetic gain using genomic prediction is probably even higher, because more information is available to reduce the limitations due to inbreeding of the sibling-based selection methods based on PBLUP, i.e. individual vs. family ranking.

## Supplementary Information

**Additional file 1: Table S1.** Summary of data for challenge test and analysis.

**Additional file 2: Table S2.** Summary of the key model parameters for Bayesian models. The file contains a summary of the posterior mean standard deviation of the key model parameters for different SNP densities and Bayesian models.

**Additional file 3: Figure S1.** Relative increase in prediction accuracy for genomic models, compared to the PBLUP model. The file contains the figure showing a relative increase in prediction accuracy for genomic models, compared to PBLUP and the pattern of heritabilities with decreasing SNP density.

## Data Availability

The data used in the study are from commercial family material. This information may be made available to non-competitive interests under conditions specified in a Data Transfer Agreement. Requests to access these datasets should be directed to Alejandro Tola Alvarez: alex@genomar.com.

## References

[1] Weimin M. Aquaculture production and trade trends: carp, tilapia and shrimp. 2017. http://www.fao.org/fi/static-media/MeetingDocuments/WorkshopAMR17/presentations/28.pdf/. Accessed 5 Sep 2019.

[2] Cai J, Zhou X, Yan X, Lucente D, Lagana C. Top 10 species groups in global aquaculture 2017. Rome: FAO Fisheries and Aquaculture Department; 2019. http://www.fao.org/3/ca5224en/ca5224en.pdf/. Accessed 5 Sep 2019.

[3] FAO. FAO Global Fishery and Aquaculture Production Statistics 1950–2017 v2019.1.0. 2019. www.fao.org/fishery/statistics/software/fishstatj/en/. Accessed 5 Sep 2019.

[4] Barroso RM, Muñoz AEP, Cai J. Social and economic performance of tilapia farming in Brazil. Rome: FAO Fisheries and Aquaculture Circular No 1181; 2019.

[5] Popma TJ, Lovshin LL (1995). Worldwide prospects for commercial production of tilapia.

[6] Amal MNA, Zamri-Saad M (2011). Streptococcosis in tilapia (*Oreochromis niloticus*): a review. Pertanika J Trop Agric Sci.

[7] Haenen O. Major bacterial diseases affecting aquaculture. http://www.fao.org/fi/static-media/MeetingDocuments/WorkshopAMR/presentations/07_Haenen.pdf. 2017. Accessed 9 Dec 2020.

[8] The fish site. Streptococcosis in tilapia: A more complex problem. 2019. https://thefishsite.com/articles/streptococcosis-in-tilapia-a-more-complex-problem/. Accessed 18 Mar 2019.

[9] Robinson JA, Meyer FP (1966). Streptococcal fish pathogen. J Bacteriol.

[10] Yanong RPE, Francis-Floyd R. Streptococcal infections of fish. Gainesville: Circular 57, School of Forest Resources and Conservation, Program in Fisheries and Aquatic, UF/IFAS Extension. 2002.

[11] Fletcher R. Streptococcus vaccine offers hope for tilapia sector. 2019. https://thefishsite.com/articles/streptococcus-vaccine-offers-hope-for-tilapia-sector/. Accessed 25 May 2020.

[12] Klesius PH, Shoemaker CA, Evans JJ. Streptococcus: a worldwide fish health problem. In Proceedings of the 8th International Symposium on Tilapia in Aquaculture: 12–14 October 2008; Cairo. 2008.

[13] Austin B, Austin DA (2012). Bacterial fish pathogens.

[14] Osman KM, Al-Maary KS, Mubarak AS, Dawoud TM, Moussa IMI, Ibrahim MDS (2017). Characterization and susceptibility of streptococci and enterococci isolated from Nile tilapia (*Oreochromis niloticus*) showing septicaemia in aquaculture and wild sites in Egypt. BMC Vet Res.

[15] Klesius P, Shoemaker C, Evans J. Vaccination: A health management practice for preventing diseases caused by streptococcus in tilapia and other cultured fish. In Proceedings of the 5^th^ International Symposium on Tilapia in Aquaculture: 3–7 September 2000; Rio de Janeiro. 2000.

[16] Evans JJ, Klesius PH, Shoemaker CA (2004). Efficacy of *Streptococcus agalactiae* (group B) vaccine in tilapia (*Oreochromis niloticus*) by intraperitoneal and bath immersion administration. Vaccine.

[17] Shoemaker C, Klesius P. Streptococcal disease problems and control: a review. In: Proceedings of the 4th International Symposium on Tilapia in Aquaculture: 9–12 November 1997; Orland. 1997.

[18] Cabello FC, Godfrey HP, Tomova A, Ivanova L, Dölz H, Millanao A (2013). Antimicrobial use in aquaculture re-examined: its relevance to antimicrobial resistance and to animal and human health. Environ Microbiol.

[19] Bishop SC, Woolliams JA (2014). Genomics and disease resistance studies in livestock. Livest Sci.

[20] Chevassus B, Dorson M (1990). Genetics of resistance to disease in fishes. Aquaculture.

[21] Beacham TD, Evelyn TPT (1992). Genetic variation in disease resistance and growth of chinook, coho, and chum salmon with respect to vibriosis, furunculosis, and bacterial kidney disease. Trans Am Fish Soc.

[22] Storset A, Strand C, Wetten M, Kjøglum S, Ramstad A (2007). Response to selection for resistance against infectious pancreatic necrosis in Atlantic salmon (*Salmo salar* L.). Aquaculture..

[23] Shoemaker CA, Lozano CA, LaFrentz BR, García JC, Soto E, Xu D-H (2017). Additive genetic variation in resistance of Nile tilapia (*Oreochromis niloticus*) to *Streptococcus iniae* and *S. agalactiae* capsular type Ib: Is genetic resistance correlated?. Aquaculture..

[24] Sukhavachana S, Poompuang S, Onming S, Luengnaruemitchai A (2019). Heritability estimates and selection response for resistance to *Streptococcus agalactiae* in red tilapia *Oreochromis* spp.. Aquaculture.

[25] LaFrentz BR, Lozano CA, Shoemaker CA, García JC, Xu D-H, Løvoll M (2016). Controlled challenge experiment demonstrates substantial additive genetic variation in resistance of Nile tilapia (*Oreochromis niloticus*) to *Streptococcus iniae*. Aquaculture.

[26] GenoMar Genetics AS. Selection for Streptococcus resistance. Oslo; 2019.

[27] Ødegård J, Moen T, Santi N, Korsvoll SA, Kjøglum S, Meuwissen THE (2014). Genomic prediction in an admixed population of Atlantic salmon (*Salmo salar*). Front Genet.

[28] Lillehammer M, Meuwissen THE, Sonesson AK (2013). A low-marker density implementation of genomic selection in aquaculture using within-family genomic breeding values. Genet Sel Evol.

[29] Joshi R, Skaarud A, de Vera M, Tola AA. Genetic parameters for commercial traits in Nile tilapia using multivariate genomic models. In: Proceedings of the 12th International Symposium on Tilapia in Aquaculture:19–20 June 2019; Chennai; 2019.

[30] Joshi R, Skaarud A, de Vera M, Alvarez AT, Odegard J (2019). Genomic prediction for commercial traits using univariate and multivariate approaches in Nile tilapia (*Oreochromis niloticus*). Aquaculture.

[31] Yoshida GM, Lhorente JP, Correa K, Soto J, Salas D, Yañez JM (2019). Genome-wide association study and cost-efficient genomic predictions for growth and fillet yield in Nile Tilapia (*Oreochromis niloticus*). G3..

[32] Joshi R, Almeida DB, da Costa AR, Skaarud A, de Pádua PU, Knutsen TM (2021). Genomic selection for resistance to Francisellosis in commercial Nile tilapia population: genetic and genomic parameters, correlation with growth rate and predictive ability. Aquaculture.

[33] Yáñez JM, Joshi R, Yoshida GM (2020). Genomics to accelerate genetic improvement in tilapia. Anim Genet.

[34] Eknath AE, Tayamen MM, Palada-de Vera MS, Danting JC, Reyes RA, Dionisio EE (1993). Genetic improvement of farmed tilapias: the growth performance of eight strains of *Oreochromis niloticus* tested in different farm environments. Aquaculture.

[35] Lin M. Statistical model comparison in genetic analysis of challenge test data on Streptococcus agalactiae resistance in Nile tilapia (*Oreochromis niloticus*). Master thesis, Norwegian University of Life Sciences; 2016.

[36] Joshi R, Arnyasi M, Lien S, Gjoen HM, Alvarez AT, Kent M (2018). Development and validation of 58K SNP-array and high-density linkage map in Nile tilapia (*O. niloticus*). Front Genet..

[37] Thermo Fisher Scientific Inc. Axiom^TM^ Analysis Suite (AxAS) v4.0 USER GUIDE. 2018. https://downloads.thermofisher.com/Affymetrix_Softwares/Axiom_Analysis_Suite_AxAS_v4.0_User_Guide.pdf/. Accessed 3 Mar 2019.

[38] Madsen P, Jensen J, Labouriau R, Christensen OF, Sahana G. DMU-a package for analyzing multivariate mixed models in quantitative genetics and genomics. In: Proceedings of the 10th World Congress on Genetics Applied to Livestock Production: 17–22 August 2014; Vancouver. 2014.

[39] Joshi R, Woolliams J, Meuwissen T, Gjøen H (2018). Maternal, dominance and additive genetic effects in Nile tilapia; influence on growth, fillet yield and body size traits. Heredity.

[40] Koivula M, Strandén I, Su G, Mäntysaari EA (2012). Different methods to calculate genomic predictions—Comparisons of BLUP at the single nucleotide polymorphism level (SNP-BLUP), BLUP at the individual level (G-BLUP), and the one-step approach (H-BLUP). J Dairy Sci.

[41] Goddard ME, Hayes BJ, Meuwissen THE (2011). Using the genomic relationship matrix to predict the accuracy of genomic selection. J Anim Breed Genet.

[42] Goddard M (2009). Genomic selection: prediction of accuracy and maximisation of long term response. Genetica.

[43] VanRaden PM, Van Tassel CP, Wiggans GR, Sonstegard TS, Schnabel RD, Taylor JF (2009). Invited review: reliability of genomic predictions for North American Holstein bulls. J Dairy Sci.

[44] Habier D, Fernando RL, Dekkers JCM (2007). The impact of genetic relationship information on genome-assisted breeding values. Genetics.

[45] VanRaden PM (2008). Efficient methods to compute genomic predictions. J Dairy Sci.

[46] Vallejo RL, Leeds TD, Gao G, Parsons JE, Martin KE, Evenhuis JP (2017). Genomic selection models double the accuracy of predicted breeding values for bacterial cold water disease resistance compared to a traditional pedigree-based model in rainbow trout aquaculture. Genet Sel Evol.

[47] Yoshida GM, Bangera R, Carvalheiro R, Correa K, Figueroa R, Lhorente JP (2018). Genomic prediction accuracy for resistance against *Piscirickettsia salmonis* in farmed rainbow trout. G3..

[48] Zeng J, De Vlaming R, Wu Y, Robinson MR, Lloyd-Jones LR, Yengo L (2018). Signatures of negative selection in the genetic architecture of human complex traits. Nat Genet.

[49] Meuwissen THE, Hayes BJ, Goddard ME (2001). Prediction of total genetic value using genome-wide dense marker maps. Genetics.

[50] Habier D, Fernando RL, Kizilkaya K, Garrick DJ (2011). Extension of the Bayesian alphabet for genomic selection. BMC Bioinform.

[51] Erbe M, Hayes BJ, Matukumalli LK, Goswami S, Bowman PJ, Reich CM (2012). Improving accuracy of genomic predictions within and between dairy cattle breeds with imputed high-density single nucleotide polymorphism panels. J Dairy Sci.

[52] de los Campos G, Hickey JM, Pong-Wong R, Daetwyler HD, Calus MPL (2013). Whole-genome regression and prediction methods applied to plant and animal breeding. Genetics..

[53] Geweke J (1991). Evaluating the accuracy of sampling-based approaches to the calculation of posterior moments.

[54] Plummer M, Best N, Cowles K, Vines K (2006). CODA: convergence diagnosis and output analysis for MCMC. R News.

[55] Purcell S, Neale B, Todd-Brown K, Thomas L, Ferreira MA, Bender D (2007). PLINK: a tool set for whole-genome association and population-based linkage analyses. Am J Hum Genet.

[56] Fischer RA (1944). Statistical methods for research workers.

[57] Conte MA, Joshi R, Moore EC, Nandamuri SP, Gammerdinger WJ, Clark FE (2019). Chromosome-scale assemblies reveal the structural evolution of African cichlid genomes. Gigascience..

[58] Chang CC, Chow CC, Tellier LCAM, Vattikuti S, Purcell SM, Lee JJ (2015). Second-generation PLINK: rising to the challenge of larger and richer datasets. Gigascience.

[59] Kaplan EL, Meier P (1958). Nonparametric estimation from incomplete observations. J Am Stat Assoc.

[60] Heringstad B, Rekaya R, Gianola D, Klemetsdal G, Weigel KA (2003). Genetic change for clinical mastitis in Norwegian cattle: a threshold model analysis. J Dairy Sci.

[61] Ødegård J, Sommer A-I, Præbel AK (2010). Heritability of resistance to viral nervous necrosis in Atlantic cod (*Gadus morhua* L.). Aquaculture..

[62] Ødegård J, Olesen I, Gjerde B, Klemetsdal G (2007). Evaluation of statistical models for genetic analysis of challenge-test data on ISA resistance in Atlantic salmon (*Salmo salar*): prediction of progeny survival. Aquaculture.

[63] Wolc A, Arango J, Settar P, Fulton JE, O’Sullivan NP, Dekkers JCM (2016). Mixture models detect large effect QTL better than GBLUP and result in more accurate and persistent predictions. J Anim Sci Biotechnol.

[64] Bangera R, Correa K, Lhorente JP, Figueroa R, Yáñez JM (2017). Genomic predictions can accelerate selection for resistance against *Piscirickettsia salmonis* in Atlantic salmon (*Salmo salar*). BMC Genom.

[65] Robledo D, Matika O, Hamilton A, Houston RD (2018). Genome-wide association and genomic selection for resistance to amoebic gill disease in Atlantic salmon. G3..

[66] Daetwyler HD, Pong-Wong R, Villanueva B, Woolliams JA (2010). The impact of genetic architecture on genome-wide evaluation methods. Genetics.

[67] Houston RD, Haley CS, Hamilton A, Guy DR, Tinch AE, Taggart JB (2008). Major quantitative trait loci affect resistance to infectious pancreatic necrosis in Atlantic salmon (*Salmo salar*). Genetics.

[68] Moen T, Baranski M, Sonesson AK, Kjøglum S (2009). Confirmation and fine-mapping of a major QTL for resistance to infectious pancreatic necrosis in Atlantic salmon (*Salmo salar*): population-level associations between markers and trait. BMC Genom.

[69] Liu S, Vallejo RL, Palti Y, Gao G, Marancik DP, Hernandez AG (2015). Identification of single nucleotide polymorphism markers associated with bacterial cold water disease resistance and spleen size in rainbow trout. Front Genet.

[70] Vela-Avitúa S, Lozano C, Bangera R, Ospina J, Rye M. Genome-wide association study for survival to *Streptococcus iniae* and *S. agalactiae* in Nile tilapia (*Oreochromis niloticus*). In Proceedings of the Fenacam’18 – XII Simpósio Internacional de Aquicultura: 13–16 November 2018; Camarao; 2018. Available from: http://abccam.com.br/wp-content/uploads/2018/11/12-Genome-wide-association-study-for-survival-to-Streptococcus-iniae-and-S.-agalactiae-in-Nile-Tilapia-Oreochromis-niloticus-Sergio-Vela.pdf/. Accessed 05 Mar 2019.

[71] Weigel KA, De Los CG, González-Recio O, Naya H, Wu XL, Long N (2009). Predictive ability of direct genomic values for lifetime net merit of Holstein sires using selected subsets of single nucleotide polymorphism markers. J Dairy Sci.

[72] Meuwissen THE (2009). Accuracy of breeding values of’unrelated’individuals predicted by dense SNP genotyping. Genet Sel Evol.

[73] Zhang Z, Ding X, Liu J, Zhang Q, de Koning DJ (2011). Accuracy of genomic prediction using low-density marker panels. J Dairy Sci.

[74] Żukowski K, Suchocki T, Gontarek A, Szyda J (2009). The impact of single nucleotide polymorphism selection on prediction of genomewide breeding values. BMC Proc.

[75] Vitezica Z, Aguilar I, Misztal I, Legarra A (2011). Bias in genomic predictions for populations under selection. Genet Res (Camb).

