# Supplementary material for: Bayesian genomic models boost prediction accuracy for survival to Streptococcus agalactiae infection in Nile tilapia (Oreochromus nilioticus)
Source: Genet Sel Evol. 2021 Apr 21;53:37. doi: 10.1186/s12711-021-00629-y (PMC8058985; doi:10.1186/s12711-021-00629-y)
Supplement: Supplementary file 1 — Additional file 1: Table S1. Summary of data for challenge test and analysis. [file 12711_2021_629_MOESM1_ESM.pdf]

Additional File 1

Bayesian genomic models boost prediction accuracy for survival to *Streptococcus agalactiae* infection in Nile tilapia (*Oreochromus nilioticus*)

Rajesh Joshi, Anders Skaaurd, Alejandro Tola Alvarez, Thomas Moen, Jørgen Ødegård

Supplementary Table 1: Summary of data for challenge test and analysis

	**Challenge test**	**Genotyping**	**Analysis after filtration**
**Number of families**	108	108	108
**Fish per family**	40<br>(15 IP+25 Cohab)	25 Cohab	~23 Cohab
**Total Fish**	4320<br>(1620 IP+2700 Cohab)	2700 Cohab	2472 cohab
**% Mortality**	60.2%	58.11%	60%
