# Supplementary material for: Bayesian genomic models boost prediction accuracy for survival to Streptococcus agalactiae infection in Nile tilapia (Oreochromus nilioticus)
Source: Genet Sel Evol. 2021 Apr 21;53:37. doi: 10.1186/s12711-021-00629-y (PMC8058985; doi:10.1186/s12711-021-00629-y)
Supplement: Supplementary file 2 — Additional file 2: Table S2. Summary of the key model parameters for Bayesian models. The file contains a summary of the posterior mean standard deviation of the key model parameters for different SNP densities and Bayesian models. [file 12711_2021_629_MOESM2_ESM.pdf]

Additional File 2

Bayesian genomic models boost prediction accuracy for survival to *Streptococcus agalactiae* infection in Nile tilapia (*Oreochromus nilioticus*)

Rajesh Joshi, Anders Skaaurd, Alejandro Tola Alvarez, Thomas Moen, Jørgen Ødegård

Supplementary Table 2: Summary of the posterior mean $^{\text{standard deviation}}$ of the key model parameters for different SNP densities and Bayesian models for *Streptococcus* resistance in Nile tilapia.

Subset (n)	Bayes	π	SNP≠0	σ^2	π_2	π_3	π_4	S
**LD0.1 (589)**	B	$0.035^{0.012}$	20^{6}					
	C	$0.127^{0.046}$	74^{26}	$1.1E\text{-}03^{3.6E\text{-}04}$				
	R	$0.171^{0.135}$	489^{80}	$2.5E\text{-}04^{1.3E\text{-}04}$	$0.152^{0.128}$	$0.278^{0.199}$	$0.399^{0.173}$	
	S	$0.777^{0.159}$	459^{93}	$1.1E\text{-}04^{5.3E\text{-}05}$				$-0.21^{0.53}$
**LD0.2 (1,544)**	B	$0.056^{0.012}$	86^{15}					
	C	$0.129^{0.038}$	198^{56}	$8.2E\text{-}04^{2.6E\text{-}04}$				
	R	$0.290^{0.194}$	1096^{300}	$4.6E\text{-}04^{3.7E\text{-}04}$	$0.269^{0.176}$	$0.214^{0.166}$	$0.227^{0.147}$	
	S	$0.353^{0.135}$	546^{209}	$6.3E\text{-}05^{3.6E\text{-}05}$				$-1.51^{0.31}$
**LD0.3 (3,384)**	B	$0.036^{0.008}$	120^{24}					
	C	$0.073^{0.028}$	247^{93}	$6.3E\text{-}04^{2.6E\text{-}04}$				
	R	$0.195^{0.146}$	2724^{493}	$1.8E\text{-}04^{2.1E\text{-}04}$	$0.251^{0.217}$	$0.193^{0.129}$	$0.361^{0.237}$	
	S	$0.203^{0.151}$	685^{512}	$6.1E\text{-}05^{4.0E\text{-}05}$				$-1.51^{0.39}$
**LD0.4 (6,229)**	B	$0.036^{0.007}$	223^{41}					
	C	$0.041^{0.018}$	256^{115}	$7.8E\text{-}04^{3.3E\text{-}04}$				
	R	$0.528^{0.276}$	2937^{1718}	$4.2E\text{-}04^{4.8E\text{-}04}$	$0.138^{0.084}$	$0.108^{0.123}$	$0.226^{0.227}$	
	S	$0.063^{0.027}$	394^{165}	$8.1E\text{-}05^{5.0E\text{-}05}$				$-1.84^{0.35}$
**LD0.5 (10,004)**	B	$0.033^{0.006}$	328^{60}					
	C	$0.041^{0.024}$	412^{236}	$5.2E\text{-}04^{3.0E\text{-}04}$				
	R	$0.338^{0.268}$	6619^{2680}	$1.7E\text{-}04^{1.4E\text{-}04}$	$0.328^{0.268}$	$0.216^{0.253}$	$0.118^{0.095}$	
	S	$0.052^{0.020}$	523^{198}	$4.1E\text{-}05^{2.7E\text{-}05}$				$-2.19^{0.33}$
**LD0.6 (14,563)**	B	$0.031^{0.006}$	445^{85}					
	C	$0.029^{0.017}$	427^{243}	$5.0E\text{-}04^{3.0E\text{-}04}$				
	R	$0.480^{0.167}$	7569^{2426}	$4.1E\text{-}05^{1.7E\text{-}05}$	$0.136^{0.088}$	$0.157^{0.136}$	$0.227^{0.093}$	
	S	$0.051^{0.027}$	742^{387}	$2.6E\text{-}05^{2.3E\text{-}05}$				$-2.42^{0.37}$
**LD0.7 (19,873)**	B	$0.032^{0.005}$	627^{105}					
	C	$0.023^{0.027}$	465^{526}	$1.3E\text{-}03^{1.1E\text{-}03}$				
	R	$0.438^{0.195}$	11171^{3878}	$9.2E\text{-}05^{3.2E\text{-}05}$	$0.237^{0.141}$	$0.276^{0.142}$	$0.050^{0.037}$	
	S	$0.033^{0.016}$	665^{314}	$3.7E\text{-}05^{4.6E\text{-}05}$				$-2.29^{0.36}$
**LD0.8 (25,693)**	B	$0.029^{0.005}$	748^{135}					
	C	$0.005^{0.001}$	117^{34}	$2.4E\text{-}03^{8.9E\text{-}04}$				
	R	$0.660^{0.108}$	8731^{2784}	$1.4E\text{-}04^{1.6E\text{-}04}$	$0.149^{0.087}$	$0.159^{0.117}$	$0.032^{0.020}$	
	S	$0.033^{0.020}$	845^{504}	$2.1E\text{-}05^{5.1E\text{-}05}$				$-2.74^{0.38}$
**LD0.9 (32,077)**	B	$0.027^{0.005}$	863^{162}					
	C	$0.003^{0.001}$	101^{26}	$3.2E\text{-}03^{1.1E\text{-}03}$				
	R	$0.506^{0.230}$	15850^{7390}	$3.0E\text{-}05^{1.1E\text{-}05}$	$0.201^{0.149}$	$0.180^{0.093}$	$0.113^{0.058}$	
	S	$0.019^{0.012}$	598^{376}	$7.5E\text{-}05^{1.7E\text{-}04}$				$-2.54^{0.57}$
**Only LG (48,871)**	B	$0.023^{0.004}$	1125^{211}					
	C	$0.001^{0.001}$	47^{34}	$2.4E\text{-}02^{8.4E\text{-}03}$				
	R	$0.558^{0.177}$	21612^{8641}	$1.4E\text{-}05^{7.0E\text{-}06}$	$0.167^{0.130}$	$0.108^{0.073}$	$0.167^{0.102}$	
	S	$0.001^{0.000}$	40^{7}	$2.4E\text{-}02^{1.5E\text{-}02}$				$-0.24^{0.52}$
**All SNPs (50,690)**	B	$0.022^{0.004}$	1131^{223}					
	C	$0.011^{0.023}$	581^{1165}	$1.9E\text{-}02^{1.2E\text{-}02}$				
	R	$0.980^{0.037}$	1003^{1867}	$2.6E\text{-}02^{1.7E\text{-}02}$	$0.009^{0.013}$	$0.002^{0.004}$	$0.009^{0.023}$	
	S	$0.020^{0.045}$	1008^{2262}	$2.0E\text{-}02^{1.8E\text{-}02}$				$-0.44^{0.75}$

Subsets LD0.1 to LD0.9 represent the SNP subsets obtained after pruning the SNPs based on LD values. For example: in subset "LD0.1" only 1 SNP in a pair or group of SNPs that were in higher LD value than 0.1 was kept;

n is the number of SNPs in each SNP subset; **π, π_2, π_3** and **π_4** are the priors of respective Bayesian models referring to the proportion of SNPs that have non-zero effects; **SNP≠0** is the number of SNPs having non-zero effects; **σ^2** is the variance of the distribution of genetic effects; **S** is the parameter describing the relationship between minor allele frequency and variance of SNP effects in BayesS;
For Bayes R, the sum of **π, π_2, π_3** and **π_4** constrained to sum to 1.
