# Supplementary material for: Bayesian genomic models boost prediction accuracy for survival to Streptococcus agalactiae infection in Nile tilapia (Oreochromus nilioticus)
Source: Genet Sel Evol. 2021 Apr 21;53:37. doi: 10.1186/s12711-021-00629-y (PMC8058985; doi:10.1186/s12711-021-00629-y)
Supplement: Supplementary file 3 — Additional file 3: Figure S1. Relative increase in prediction accuracy for genomic models, compared to the PBLUP model. The file contains the figure showing a relative increase in prediction accuracy for genomic models, compared to PBLUP and the pattern of heritabilities with decreasing SNP density. [file 12711_2021_629_MOESM3_ESM.pdf]

Additional File 3

Bayesian genomic models boost prediction accuracy for survival to *Streptococcus agalactiae* infection in Nile tilapia (*Oreochromus nilioticus*)

Rajesh Joshi, Anders Skaaurd, Alejandro Tola Alvarez, Thomas Moen, Jørgen Ødegård

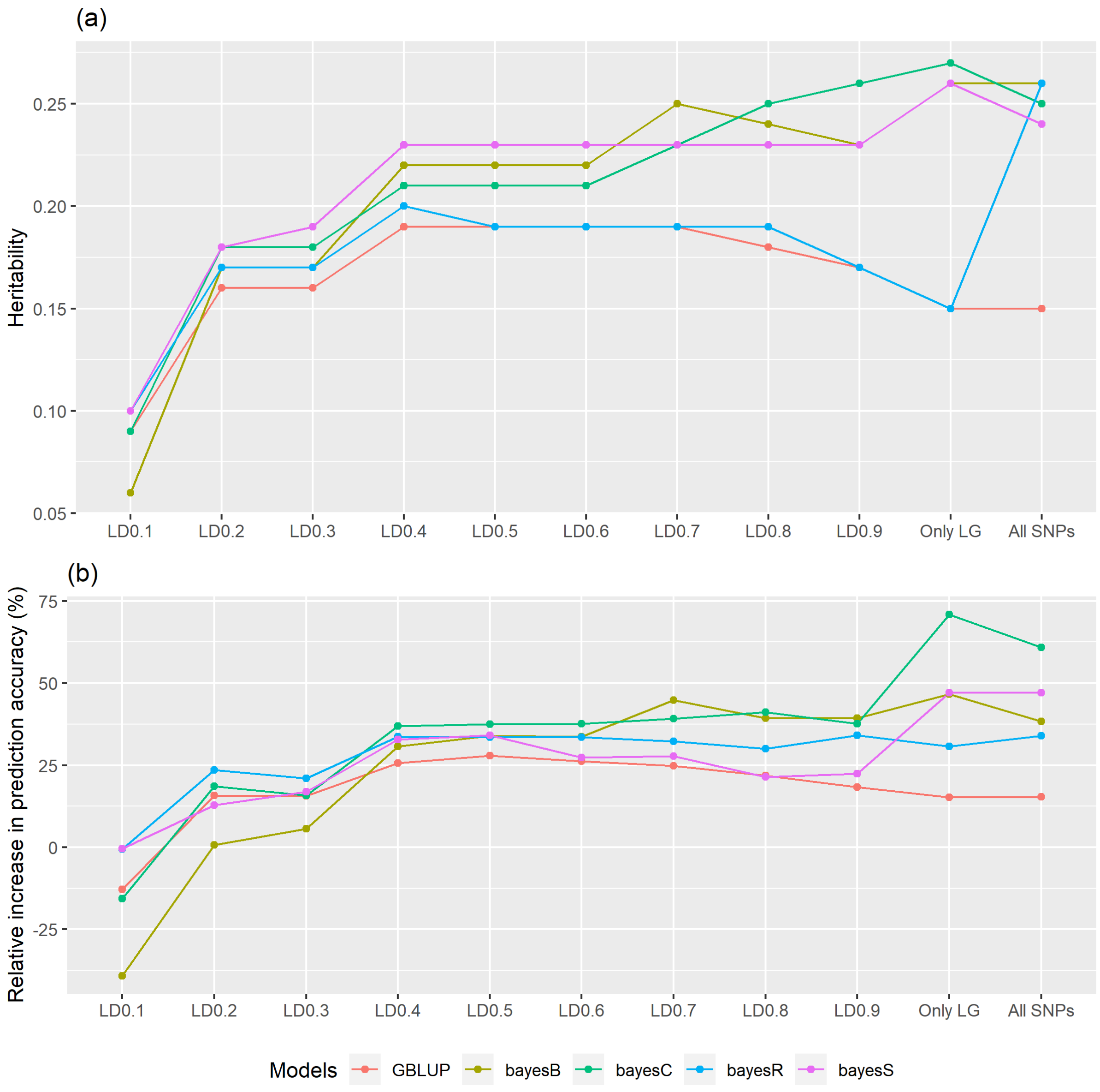

Supplementary Figure 1: (a) First figure shows the pattern of heritabilities with decreasing SNP density (from right to left) for different models. (b) The second figure shows the pattern of the relative increase in prediction accuracy (%) compared to PBLUP with decreasing SNP density (from right to left) for different models.
